# A novel site comes into sight

**DOI:** 10.7554/eLife.01680

**Published:** 2013-12-03

**Authors:** Yu Wang, Andrew P McMahon

**Affiliations:** 1 **Yu Wang** is at the State Key Laboratory of Reproductive Biology, Institute of Zoology, Chinese Academy of Sciences, Beijing, China and the School of Pharmacy, University of Wisconsin, Madison, United Statesyuwang@post.harvard.edu; 2 **Andrew P McMahon** is at the Department of Stem Cell Biology and Regenerative Medicine, Eli and Edythe Broad-California Institute for Regenerative Medicine Center for Regenerative Medicine and Stem Cell Research, Keck School of Medicine of the University of Southern California, Los Angeles, United States

**Keywords:** Hedgehog signaling, oxysterol, smoothened, cysteine rich domain, Zebrafish

## Abstract

Oxysterols modulate the Hedgehog signalling pathway by binding a novel site on the membrane protein Smoothened, which may offer new options for the treatment of cancers linked to this pathway.

**Related research article** Nachtergaele S, Whalen DM, Mydock LK, Zhao Z, Malinauskas T, Krishnan K, Ingham PW, Covey DF, Siebold C, Rohatgi R. 2013. Structure and function of the Smoothened extracellular domain in vertebrate hedgehog signalling. *eLife* **2**:e01340. doi: 10.7554/eLife.01340

**Image** The oxysterol binding site (purple) in zebrafish Smoothened

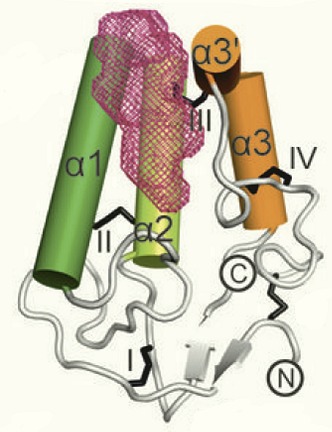

The Hedgehog signalling pathway plays a central role in development and the maintenance of normal tissue function in adults. It has also been implicated in degenerative diseases and a multitude of cancers. A great deal of effort has been invested in designing drugs that target this pathway (Dellovade et al., 2006; Rubin and de Sauvage, 2006), and after more than a decade of work in both academic laboratories and pharmaceutical companies, several anticancer drugs that regulate the Hedgehog pathway have now entered the clinic. Most of these drugs target a membrane protein called Smoothened.

Now, in *eLife*, Rajat Rohatgi at Stanford University School of Medicine and co-workers in the US, UK and Singapore—including Sigrid Nachtergaele (Stanford) and Daniel Whalen (Oxford) as joint first authors—provide new insights into the regulation of Smoothened by oxysterols, a class of naturally occurring compounds, and suggest new options for the pharmacological modulation of Hedgehog signalling (Nachtergaele et al., 2013). Similar results have also been reported in *Nature Chemical Biology* by Daniel Nedelcu of Harvard Medical School and co-workers (Nedelcu et al., 2013), and in *Developmental Cell* by Benjamin Myers of Stanford University School of Medicine and co-workers (Myers et al., 2013).

Hedgehog signalling at the cell membrane involves two essential components: Patched1, which is a receptor for the Hedgehog signalling molecule, and Smoothened. In the absence of Hedgehog, Patched1 inhibits Smoothened. However, when Hedeghog binds to Patched1, Smoothened gets activated (Briscoe and Therond, 2013). There is now a general consensus that the interaction between Patched1 and Smoothened is mediated by a small molecule (or molecules) that is regulated by Patched1, rather than by direct physical interactions between the two (Taipale et al., 2002). This might explain why Smoothened has emerged as the most ‘druggable’ target in the Hedgehog pathway (Rubin and de Sauvage, 2006).

To date, the best candidates to emerge as the endogenous regulators of Smoothened activity are oxysterols, which are naturally occurring compounds produced through the oxidation of cholesterol (Corcoran and Scott, 2006; Dwyer et al., 2007). Of these, one of the most potent is 20(S)-hydroxycholesterol (20(S)-OHC). Previous work has shown that oxysterols modulate Smoothened in an allosteric manner; that is, in contrast to several other known modulators of this protein, oxysterols bind at a distinct site (Nachtergaele et al., 2012). The three new papers give detailed insights into the mechanism of action of oxysterol, and lend support to a model based on allosteric interactions.

**Figure 1.**
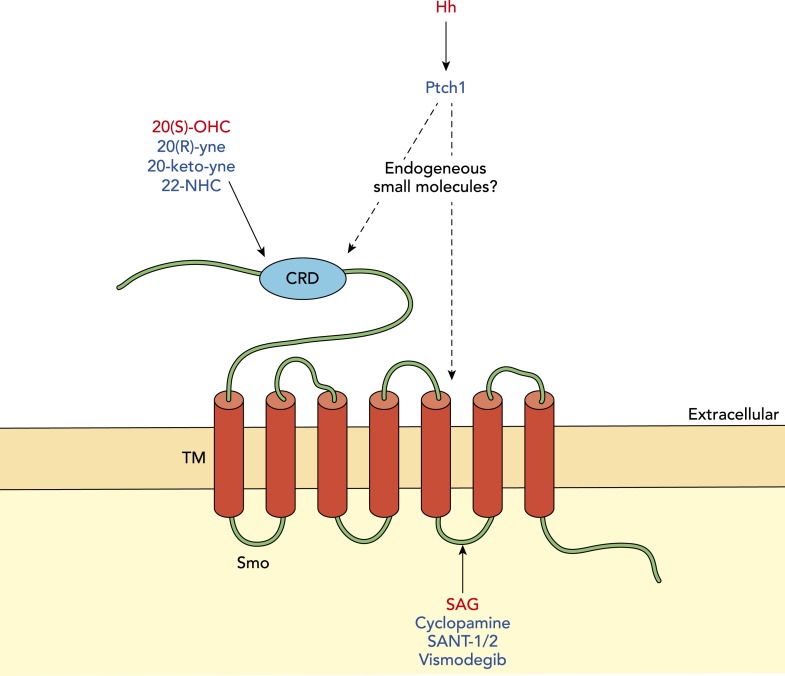
Modulation of Smoothened in vertebrates. The signalling molecule Hedgehog (Hh) acts via a receptor called Patched1 (Ptch1) to modulate the activity of another membrane protein called Smoothened (Smo). Activators of Smo activity are shown in red and inhibitors are shown in blue. Oxysterols (such as 20(S)-OHC) can activate Smo by binding its cysteine-rich domain (CRD), while various synthetic molecules (such as 20(R)-yne, 20-keto-yne and 22-NHC) can inhibit Smo by binding the CRD; the small molecule Smoothened agonist (SAG) can increase Smo activity by interacting with its membrane-bound region, while cyclopamine and other chemicals (shown in blue) can decrease Smo activity by interacting with this region. The endogenous small molecule(s) that mediate the inhibition of Smoothened by Patched1 remain unknown.

Smoothened shares several structural features with a family of receptors called Frizzled, which bind a signalling molecule called Wnt. In particular, both possess a cysteine-rich domain at their N-terminus. The fact that this region of Frizzled binds a palmitoyl group that is present on Wnt ligands (Janda et al., 2012) suggested that oxysterols may likewise interact with the cysteine-rich domain in Smoothened; this has now been confirmed experimentally by the latest work from the groups at Stanford and Harvard. A variety of small molecules, including clinically relevant compounds that are known to interact with a cavity embedded in the transmembrane part of the protein (Wang et al., 2013), did not compete with 20(S)-OHC for binding Smoothened. Thus, it is clear that there are separate binding events for oxysterols and these other compounds.

With the aid of the crystal structure of the cysteine-rich domain in zebrafish Smoothened, Nachtergaele et al. used computational modelling to map oxysterol binding to a hydrophobic groove on the surface of this domain. This interaction had been predicted by Myers et al. and Nedelcu et al. using in silico modelling. The cysteine-rich domain is sufficient for binding oxysterol, but this property is lost when key amino acid residues within the domain are mutated.

The initiation of Hedgehog signalling depends on Smoothened building up in a cellular structure called the primary cilium (Goetz and Anderson, 2010). The two Stanford groups and the Harvard group find that Smoothened variants with a mutated oxysterol binding site show decreased translocation to the primary cilium and decreased pathway activity in response to 20(S)-OHC. Furthermore, mutant versions of Smoothened that lack the cysteine-rich domain do not respond to 20(S)-OHC, but show a response that is similar to the response of wild-type Smoothened to molecules that interact with the transmembrane domain. Together the data provide compelling evidence that oxysterols interact with the cysteine-rich domain to modulate Smoothened activity.

These findings raise two key questions: is 20(S)-OHC the endogenous small molecule that mediates the regulation of Smoothened by Patched1? And is this the major regulatory input controlling Smoothened activity?

The mutant lacking the cysteine-rich domain exhibits a higher basal level of signalling than intact Smoothened, suggesting that the cysteine-rich domain suppresses Smoothened activity (Aanstad et al., 2009; Myers et al., 2013; Nachtergaele et al., 2013; Nedelcu et al., 2013). However, when Patched1 and the mutant version of Smoothened are expressed in cells together, the basal activity of the mutant is diminished (Myers et al., 2013). This indicates that the mutant version is still subject to regulation by Patched1. Thus the cysteine-rich domain may provide tonic control of Smoothened activity, whereas Patched1-dependent processes regulate Smoothened via a site separate from the domain. Myers et al. also discovered that a cell line that responds to Hedgehog contains no endogenous 20(S)-OHC, suggesting that this oxysterol may not be essential for the regulation of Smoothened. Moreover, the same oxysterols do not bind Smoothened in *Drosophila*, highlighting an evolutionary divergence in regulation of the Hedgehog pathway within the animal kingdom (Nachtergaele et al., 2013; Nedelcu et al., 2013).

Depleting sterols in cells diminishes the accumulation of Smoothened in the primary cilium, and activation of the Hedgehog pathway. The observation that the Smoothened lacking the cysteine-rich domain is still sensitive to sterol depletion suggests that these compounds may have additional roles mediated through interactions outside the domain (Myers et al., 2013). The fact that the response to Hedgehog is dampened in mutants suggests that, in vertebrates, the binding of oxysterols to the cysteine-rich domain is required for maximal activity of Smoothened (Aanstad et al., 2009; Myers et al., 2013; Nachtergaele et al., 2013; Nedelcu et al., 2013).

Naturally occurring small molecule regulators of biochemical pathways can be used to guide the development of synthetic compounds that modulate the same pathways. For example, cyclopamine, a Smoothened antagonist produced by certain plants, inspired the development of the experimental anticancer drug, saridegib (Tremblay et al., 2009). The studies discussed here reveal several oxysterol analogues that inhibit the activity of the Hedgehog pathway by competing with 20(S)-OHC for access to the cysteine-rich domain in Smoothened (Nachtergaele et al., 2013; Nedelcu et al., 2013). Two of these compounds inhibit drug-resistant variants of Smoothened found in cancer patients (Nachtergaele et al., 2013). This raises the possibility of designing next generation drugs based on a sterol scaffold, and of using treatment regimes consisting of multiple drugs to target distinct sites on the Smoothened protein.

The three new studies provide a detailed picture of the role of oxysterol in the regulation of Smoothend activity, though several important questions remain. If oxysterols are not the major regulator of Smoothened function, what is? Are there multiple modulators with distinct mechanisms that still await discovery? How does Patched1 control Smoothened? This does not appear to be via oxysterols, but is presumbly through a yet-to-be-identified major control factor (or factors). Answering these questions will open up still more possibilities for pharmacological control of the Hedgehog pathway.
